# A phase I/II study on intracerebroventricular tralesinidase alfa in patients with Sanfilippo syndrome type B

**DOI:** 10.1172/JCI165076

**Published:** 2023-01-17

**Authors:** Nicole Muschol, Anja Koehn, Katharina von Cossel, Ilyas Okur, Fatih Ezgu, Paul Harmatz, Maria J. de Castro Lopez, Maria Luz Couce, Shuan-Pei Lin, Spyros Batzios, Maureen Cleary, Martha Solano, Igor Nestrasil, Brian Kaufman, Adam J. Shaywitz, Stephen M. Maricich, Bernice Kuca, Joseph Kovalchin, Eric Zanelli

**Affiliations:** 1 University Medical Center Hamburg-Eppendorf, International Center for Lysosomal Disorders (ICLD), Hamburg, Germany.; 2 Gazi University Faculty of Medicine, Departments of Pediatric Metabolism and Genetics, Ankara, Turkey.; 3 UCSF Benioff Children’s Hospital Oakland, Oakland, California, USA.; 4 Hospital Clínico Universitario de Santiago, University of Santiago de Compostela, IDIS, CIBERER, MetabERN, A Coruña, Spain.; 5 Mackay Memorial Hospital, Taipei, Taiwan.; 6 Great Ormond Street Hospital, London, United Kingdom.; 7 Fundación Cardio Infantil, Bogotá, Colombia.; 8 Division of Clinical Behavioral Neuroscience, Department of Pediatrics, and Masonic Institute for the Developing Brain, University of Minnesota, Minneapolis, Minnesota, USA.; 9 CLB Consulting, Falls of Neuse, Raleigh, North Carolina, USA.; 10 BioMarin Pharmaceutical Inc., Novato, California, USA.; 11 Allievex Corporation, Marblehead, Massachusetts, USA.

**Keywords:** Neuroscience, Neurological disorders

## Abstract

**Background:**

Sanfilippo type B is a mucopolysaccharidosis (MPS) with a major neuronopathic component characterized by heparan sulfate (HS) accumulation due to mutations in the *NAGLU* gene encoding alfa-*N*-acetyl-glucosaminidase. Enzyme replacement therapy for neuronopathic MPS requires efficient enzyme delivery throughout the brain in order to normalize HS levels, prevent brain atrophy, and potentially delay cognitive decline.

**Methods:**

In this phase I/II open-label study, patients with MPS type IIIB (*n* = 22) were treated with tralesinidase alfa administered i.c.v. The patients were monitored for drug exposure; total HS and HS nonreducing end (HS-NRE) levels in both cerebrospinal fluid (CSF) and plasma; anti-drug antibody response; brain, spleen, and liver volumes as measured by MRI; and cognitive development as measured by age-equivalent (AEq) scores.

**Results:**

In the Part 1 dose escalation (30, 100, and 300 mg) phase, a 300 mg dose of tralesinidase alfa was necessary to achieve normalization of HS and HS-NRE levels in the CSF and plasma. In Part 2, 300 mg tralesinidase alfa sustained HS and HS-NRE normalization in the CSF and stabilized cortical gray matter volume (CGMV) over 48 weeks of treatment. Resolution of hepatomegaly and a reduction in spleen volume were observed in most patients. Significant correlations were also established between the change in cognitive AEq score and plasma drug exposure, plasma HS-NRE levels, and CGMV.

**Conclusion:**

Administration of tralesinidase alfa i.c.v. effectively normalized HS and HS-NRE levels as a prerequisite for clinical efficacy. Peripheral drug exposure data suggest a role for the glymphatic system in altering tralesinidase alfa efficacy.

**Trial registration:**

Clinicaltrials.gov NCT02754076.

**FUNDING:**

BioMarin Pharmaceutical Inc. and Allievex Corporation.

## Introduction

Lysosomal storage diseases (LSDs) are a family of genetic disorders affecting the breakdown and recycling of complex molecules within the lysosomes (1). The lysosomal compartment contains more than 60 hydrolases including enzymes involved in the degradation of glycosaminoglycans (GAGs). LSDs associated with genetic deficiencies in GAG catabolism are regrouped under the term of mucopolysaccharidoses (MPS) (2, 3). MPS type III, or Sanfilippo syndrome, is a hereditary disorder characterized by the accumulation of heparan sulfate (HS), which leads to brain atrophy, developmental delay, behavioral disturbances, dementia, and a life expectancy of less than 20 years of age in most cases (4).

Sanfilippo syndrome, or MPS type III, is categorized into 4 subtypes, A–D, according to defects in genes coding for 4 different enzymes. Sanfilippo syndrome type B (MPS IIIB; Online Mendelian Inheritance in Man [OMIM] #252920) is specifically due to mutations in a gene coding for alfa-*N*-acetyl-glucosaminidase (*NAGLU*; EC 3.2.1.50) (5). In a recently published article that characterizes the natural history of individuals with MPS IIIB (6), it was established that the spectrum of disease severity in individuals with MPS IIIB based on cognitive and adaptive behavior decline and cortical gray matter atrophy represents a single continuum with predicted trajectories (6). Although a few individuals have a genetically defined attenuated phenotype, the majority of patients with MPS IIIB achieve an apex on both cognitive and adaptive behavior scales between 3 and 6 years of age, as demonstrated by age-equivalent (AEq) scores. Development quotients (DQs) for both cognition and adaptive behavior follow a linear trajectory by which individuals reach a nadir by 8 years of age on average and by 13.5 years at the latest. At baseline, all individuals tested had HS and HS nonreducing end (HS-NRE) levels in cerebrospinal fluid (CSF) and plasma above the normal range as well as signs of hepatomegaly (6).

Endogenous NAGLU contains mannose-6-phosphate (M6P) residues, which enable receptor-mediated endocytosis and targeting to the lysosome where HS is degraded (7). Unfortunately, recombinant NAGLU expressed in CHO cells is not effectively taken up by lysosomes because it contains little or no M6-P. To improve on its uptake by human cells, NAGLU was fused to insulin-like growth factor-2 (IGF2) so that the resulting fusion protein, tralesinidase alfa, could be efficiently captured through the IGF2-binding site of cation-independent M6P receptor (CI-MPR) (8). Animal studies in mice, dogs, and nonhuman primates have shown that i.c.v. delivery of tralesinidase alfa can bypass the blood-brain barrier (BBB), result in normalization of total HS and disease-specific HS-NRE, and reduce disease-associated markers in brain tissues (9, 10). In both mouse and dog models, the lysosome size decreased with tralesinidase alfa treatment, demonstrating that it was effectively clearing HS from the targeted lysosome. Additional work in a dog model of MPS IIIB has shown that tralesinidase alfa–mediated reduction in HS and HS-NRE in both CSF and brain tissue leads to improvement in a T-maze learning task (11).

The objectives of this study were to evaluate the safety, tolerability, and efficacy of tralesinidase alfa administered via an implanted i.c.v. device to patients with MPS IIIB. Here, we report the pharmacokinetics (PK) and pharmacodynamics (PD) results of our clinical investigation of tralesinidase alfa in the treatment of children with MPS IIIB over a 48-week period. Overall, the current study highlights the ability of tralesinidase alfa to alter the natural course of MPS IIIB and suggests that this positive effect may lead to meaningful clinical benefits.

## Results

### Patients’ characteristics and adverse events.

**Figure 1:**
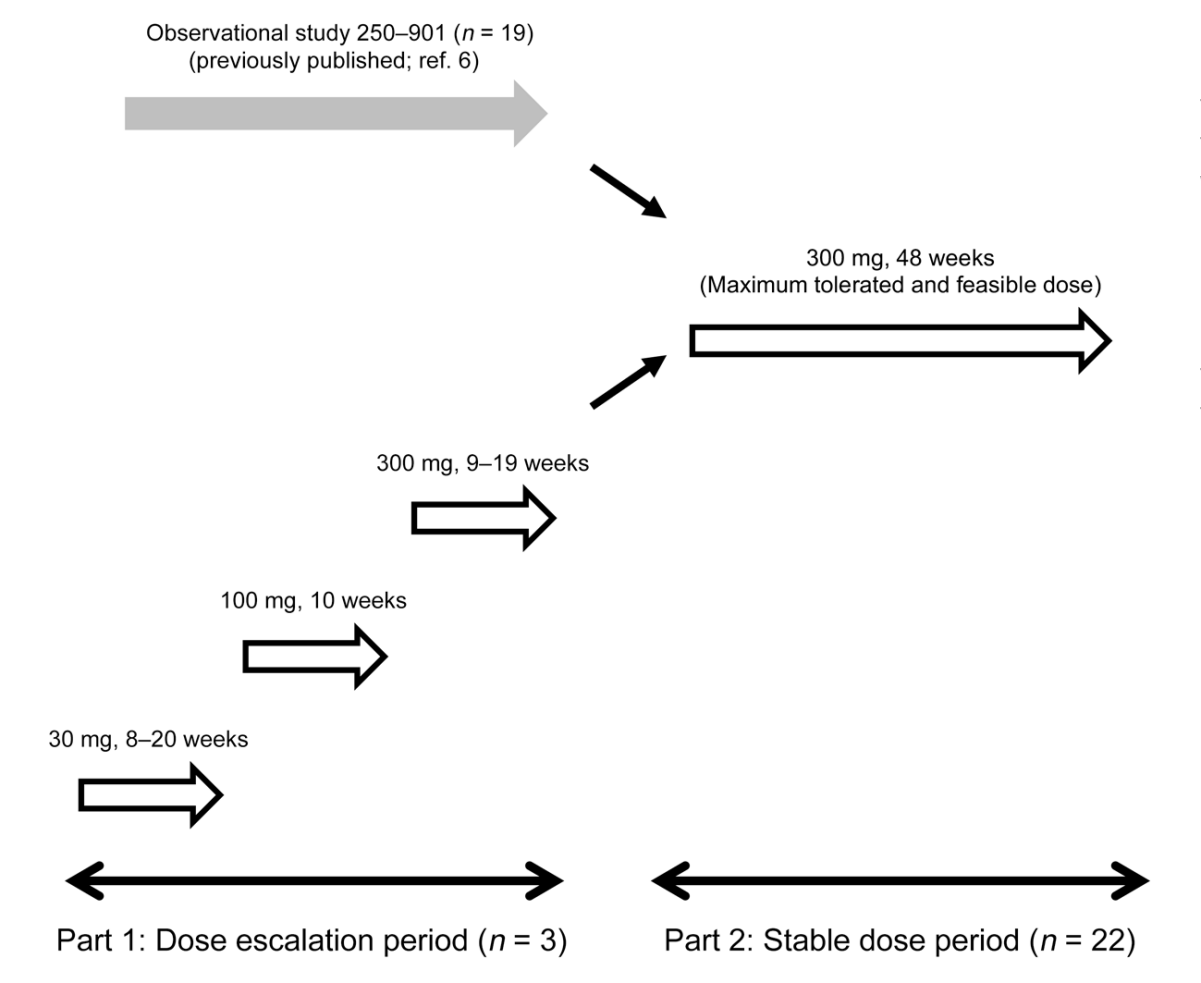
Flow diagram of Study 250-201. In Part 1, 3 patients, i.e., 9001, 9002, and 9003, were recruited and treated with escalating doses of 30, 100, and 300 mg tralesinidase alfa as described in Results. These 3 patients were eventually recruited to Part 2 of the study and treated for an additional 48 weeks. In addition, 19 patients, i.e., patients 9004–9015 and 9017–9023, previously observed in our natural history Study 250-901 (6), were treated with 300 mg tralesinidase alfa. One patient, 9016, withdrew from the trial prior to the first drug administration. In all cases, treatment was done weekly through i.c.v. administration.

Interventional Study 250-201 was divided into Part 1 and Part 2 (Figure 1). Part 1 was a dose escalation study in which 3 individuals, i.e., patients 9001, 9002, and 9003, received weekly doses of 30, 100, and 300 mg (maximum feasible dose) of tralesinidase alfa administered i.c.v. into the lateral ventricle through an implanted i.c.v. device, such as the Ommaya reservoir, and a catheter. In Part 2, twenty-two patients were scheduled to receive 48 weekly 30 mg doses of tralesinidase alfa through a similarly implanted device; 3 of these 22 individuals were the Part 1 patients who transitioned to Part 2. Nineteen individuals in Study 250-201 were previously recruited to observational Study 250-901 as previously reported (6).

Patient 9001 in Study 250-201 Part 1 received 28 weekly doses at 30 mg of tralesinidase alfa, followed by 10 doses at 100 mg, and then 11 doses at 300 mg before being recruited to Study 250-201 Part 2. Patient 9002 received 8, 10, and 19 doses of 30, 100, and 300 mg tralesinidase alfa, respectively, before being recruited to Study 250-201 Part 2, whereas patient 9003 received 20, 10, and 9 doses of 30, 100, and a 300 mg dose of tralesinidase alfa in Study 250-201 Part 1.

Characteristics of the participants in Study 250-201 Part 2 are summarized in Table 1. Thirteen males and 9 females with MPS IIIB were recruited. The average age at diagnosis was 33 months, and the average age at the time of first dosing with tralesinidase alfa was 60 months. The average cognitive DQ score at baseline was 55 for an AEq of 30 months, with DQ being defined as the AEq divided by age and multiplied by 100. Treatment compliance for Study 250-201 Part 2 was 90% (median, 98%) for 48 doses of tralesinidase alfa scheduled to be delivered over 48 weeks of weekly i.c.v. administrations. Two patients received only 17% and 57%, respectively, of the expected doses of tralesinidase alfa (Table 1). The ethnicity of the participants is indicated in Table 1 but was not taken into account in our analyses.

No deaths occurred during the course of the study. One participant discontinued the study following a serious adverse event (SAE) of subdural hygroma associated with increased intracranial pressure occurring after the seventh dose of tralesinidase alfa. This event was considered common terminology criteria for adverse events (CTCAE) grade 3 and was assessed as unrelated to the study drug or the device by the site’s principal investigator. Clinical symptoms (headache, vomiting, listlessness) resolved within 24 hours with medical management. In total, this patient received only 8 doses of tralesinidase alfa. The most common treatment-emergent adverse events (TEAEs) included vomiting, pyrexia, upper respiratory tract infection, headache, and CSF pleocytosis. SAEs assessed by investigators to be related to the study drug were CSF pleocytosis, vomiting, angioedema, fluctuating consciousness, and pyrexia. SAEs assessed by investigators to be related to the i.c.v. device were infection, device malfunction, CSF leakage, and wound infection. Overall, these TEAEs and SAEs were consistent with known complications of enzyme replacement therapy (ERT), i.c.v. devices, and/or neurodegenerative disease in pediatric populations.

Eight hypersensitivity events were reported for 5 patients, 5 events of rash, and 1 event each of angioedema, choking, and maculopapular rash. Blood samples for total IgE, C4, serum tryptase, and drug-specific IgE did not show evidence of anaphylactic reaction in any of these 5 patients for any of the hypersensitivity events observed.

### Tralesinidase alfa PK in CSF and plasma.

Table 2 summarizes the average drug exposure in CSF and plasma achieved after a single i.c.v. administration of 30 mg (*n* = 2 participants, Study 250-201 Part 1), 100 mg (3 participants, Study 250-201 Part 1), and 300 mg tralesinidase alfa (17 participants, Study 250-201 Part 2). Analysis of the PK data is focused on the 300 mg dose in 17 participants from Study 250-201 Part 2, who were not previously exposed to tralesinidase alfa.

Total CSF exposures, i.e., AUC_0–last_, achieved for patients 9002 and 9003 after a single dose of 30 mg tralesinidase alfa, were 1,130,000 and 1,620,000 ng/mL/h, for a maximum concentration (C_max_) of 403,000 and 470,000 ng/mL, measured 1 hour after drug administration, respectively. The average CSF AUC_0–last_ and C_max_ were 19,100,000 ng/mL/h and 3,440,000 ng/mL (median of 15,400,000 ng/mL/h and 2,900,000 ng/mL), respectively, achieved for patients (*n* = 17) receiving the first i.c.v. dose of tralesinidase alfa (300 mg) in Study 250-201 Part 2 (Table 2); the lowest CSF exposure (AUC_0–last_) was 5,050,000 ng/mL/h and the highest was 49,800,000 ng/mL/h. The estimated average *t*_1/2_ and *t*_last_ were 5 hours and 68 hours, respectively, suggesting that tralesinidase alfa was efficiently distributed throughout the brain and quickly absorbed via the CI-MPR, as intended. The average AUC_0–last_ and C_max_ appeared to scale linearly with the dose, suggesting that the drug exposure achieved in CSF of patients with MPS IIIB was dose proportional (Table 2).

**Figure 2:**
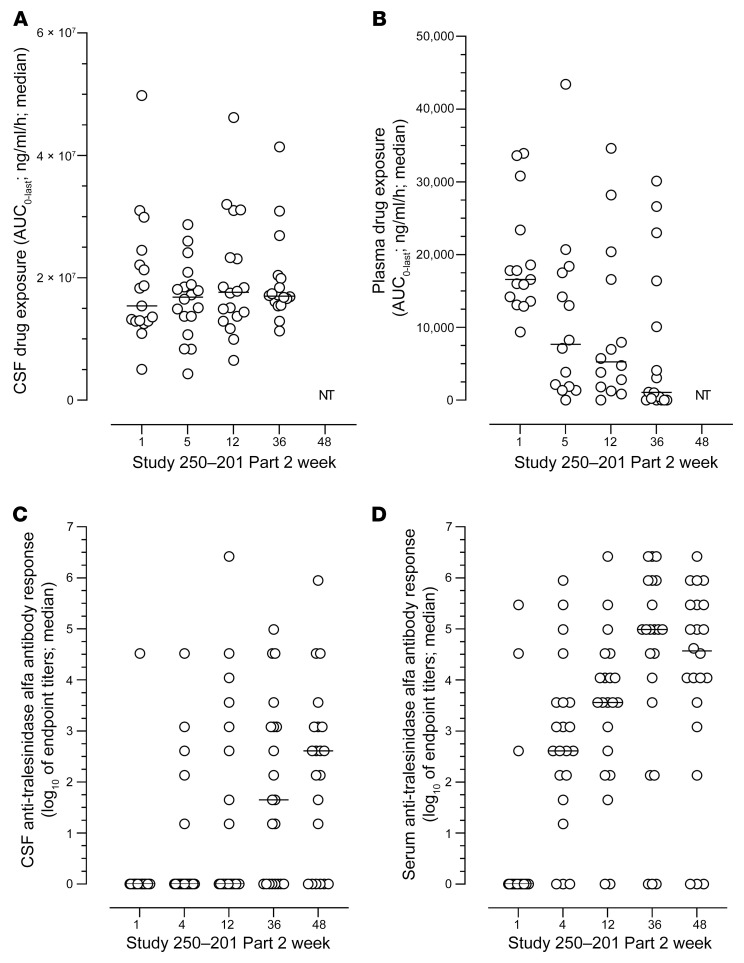
Drug exposure in plasma and CSF and anti-drug antibody response in serum and CSF. (**A**) Patients were treated i.c.v. weekly from weeks 1 to 48 (*n* = 22). Total exposure in CSF was calculated as the AUC_0–last_ in samples collected 0.5, 4, 10, 24, 48, 72, 96, and 168 hours after drug administration at week 1 (baseline) and at weeks 5, 12, and 36 of Study 250-201 Part 2. (**B**) similarly, total exposure in serum was calculated as the AUC_0–last_ at weeks 1 (baseline), 5, 12, and 36 of Study 250-201 Part 2. Anti-drug antibodies were measured in CSF (**C**) and serum (**D**) at week 1 (baseline) and at weeks 4, 12, 36, and 48 of Study 250-201. Quantification of tralesinidase alfa and titration of anti-drug antibody response were done as described in Methods. NT, not tested (i.e., samples were not collected for PK analysis at week 48). Data are presented as scattered plots with median values.

The kinetics of the drug exposure in both CSF and plasma was analyzed for 20 of 22 participants in Study 250-201 Part 2 at weeks 1, 5, 12, and 36 (Figure 2, A and B), along with anti-drug antibodies (ADAs) in the CSF and serum at weeks 1, 4, 12, 36, and 48 (Figure 2, C and D); PK data were not available for 2 of the patients. AUC_0–last_ values remained very constant over time in the CSF, with mean values of 19.1, 16.4, 19.9, and 19.3 mg/mL/h for median values of 15.4, 16.9, 17.7, and 17.0 mg/mL/h at weeks 1, 5, 12, and 36, respectively (Figure 2A). Seven days after administration, little to no tralesinidase alfa was detectable in the CSF prior to the subsequent dose; this observation, along with the fact that tralesinidase alfa did not increase during the course of the study, demonstrates that tralesinidase alfa did not accumulate over time with weekly dosing frequency.

Plasma exposure (AUC_0–last_) significantly decreased over time from a mean value of 18,800 ng/mL/h at week 1 to 10,900 ng/mL/h at week 5, to 9,700 ng/mL/h at week 12, and to 7,260 ng/mL/h at week 36 (Figure 2B; *P* = 0.05, 0.05, and 0.01 comparing data for weeks 5, 12, and 36 with data for week 1, respectively, using 1-way ANOVA with Dunnett’s multiple-comparison test). At week 1, drug exposure was, on average, 1,000-fold lower in plasma compared with exposure values in CSF (range, 300–3,300) and dropped to 22,000-fold lower by week 36 (range, 500–170,000). Of note, decreases in drug exposure in plasma were not substantial from week 5 to week 12 or from week 12 to week 36. The estimated *t*_max_ in plasma was 7.5 hours at week 1 (*n* = 16) and remained approximately 10 hours for treated patients with quantifiable drug exposure in plasma at weeks 5, 12, and 36, i.e., an AUC_0–last_ above 10,000 ng/mL/h. For the other participants, exposure was too low to estimate the C_max_ and *t*_max_.

### Immunogenicity.

The ADA response was monitored in both CSF and serum during the course of Study 250-201 Part 2 (Figure 2, C and D). Total ADA titers were measured along with specific anti-IGF1 and anti-IGF2 antibodies. Serum biochemistry showed no evidence of abnormal levels of endogenous IGF1 or IGF2, or any signs of hypoglycemia in patients enrolled in Study 250-201. Therefore, anti-IGF1 and anti-IGF2 antibody responses will not be discussed further, as they did not provide additional information beyond the findings with total anti–tralesinidase alfa antibodies.

The only 3 patients with ADA in serum at week 1 were those who had already received tralesinidase alfa treatment in Study 250-201 Part 1 (Figure 2D). In all participants, ADA titers were higher in serum than in CSF by Study 250-201 Part 2 completion (week 48). Three patients had no detectable ADAs in serum, and 6 patients had no ADAs in CSF. Only 3 patients had ADA endpoint titers above 1:3,645 (log_10_ = 3.56) in CSF at weeks 36 and 48 (Figure 2C). Conversely, the median serum ADA titers went from 1:405 (log_10_ = 2.61) at week 4 to 1:3,645 (log_10_ = 3.56), 1:98,415 (log_10_ = 4.99), and 1:37,345 (log_10_ = 4.57) at weeks 12, 36, and 48, respectively (Figure 2D), suggesting that by week 36, ADA titers had reached an apex and might stabilize or decrease beyond this time point. The presence of drug in analyzed samples did not confound ADA titration, since trough levels prior to drug administration were in the 4–7 ng/mL range on average and often below the level of quantification (data not shown).

The largest drop in plasma drug exposure occurred between week 1 and week 5 of Study 250-201 Part 2 (h2B), when the median ADA titer in serum was only 1:405 at week 4. In vitro cell-based studies suggested that antibodies detected in ADA-positive CSF samples were not able to neutralize the uptake of tralesinidase alfa (titers of neutralizing antibodies were <1:100 in ADA-positive CSF samples).

### Tralesinidase alfa 300 mg normalizes HS and HS-NRE.

**Figure 3:**
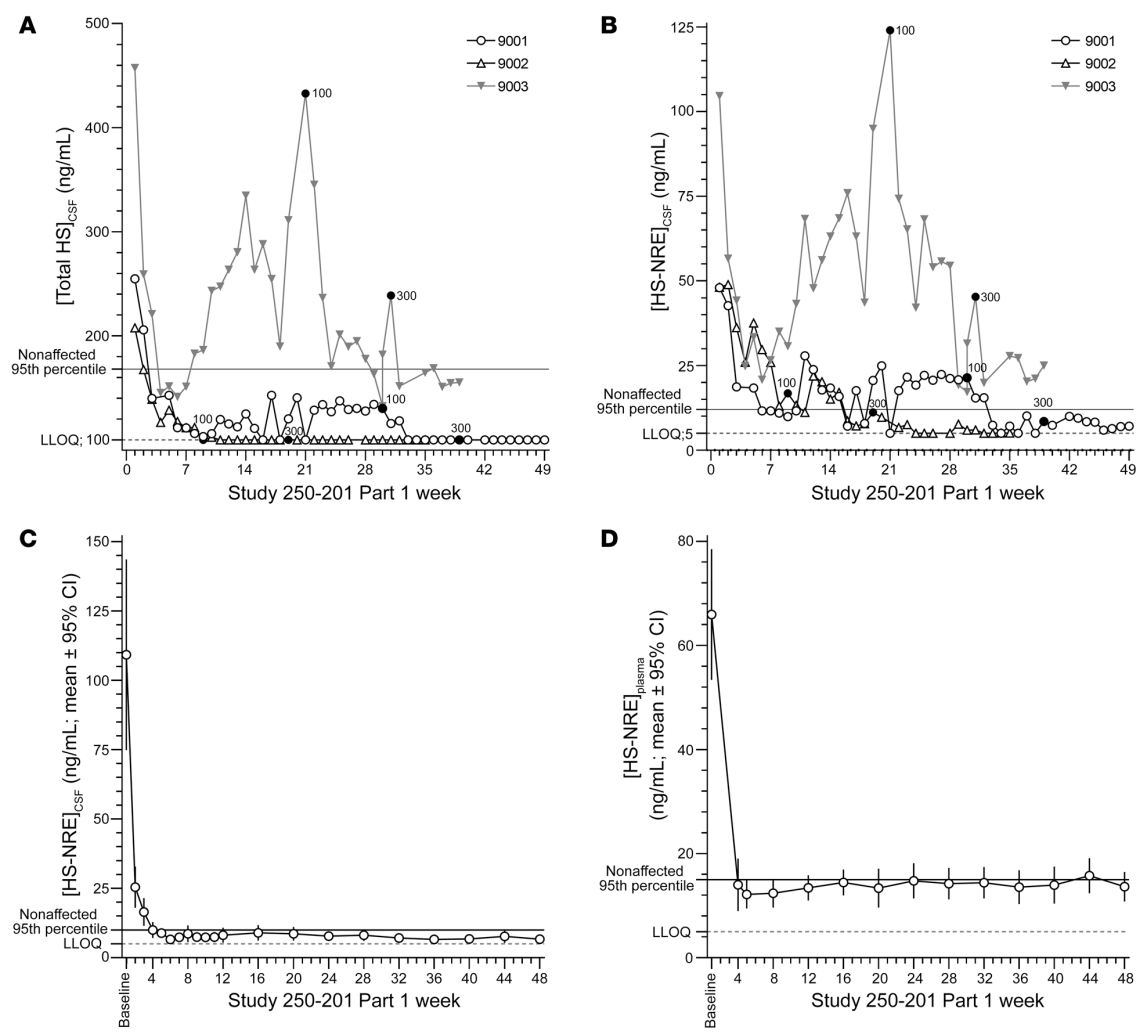
A 300 mg dose of tralesinidase alfa administered weekly i.c.v. normalizes total HS and HS-NRE levels in the CSF and plasma. In Study 250-201 Part 1, three patients, i.e., 9001, 9002, and 9003, were treated with 30, 100, or 300 mg tralesinidase alfa weekly. Total HS (**A**) and HS-NRE (**B**) concentrations were quantified weekly in the CSF of treated patients using a Sensi-Pro assay as described in Methods. Black dots labeled 100 or 300 indicate the weeks when treatment increased for each patient from 30 to 100 mg or from 100 to 300 mg. In Study 250-201 Part 2, the patients (*n* = 22) were treated weekly for 48 weeks with 300 mg tralesinidase alfa. HS-NRE was quantified in the CSF (**C**) and plasma (**D**) of treated patients weekly or at least every 4 weeks using the Sensi-Pro assay as described in Methods. Two patients were excluded from the data in **C** and **D** because they received only 17% and 57%, respectively, of the tralesinidase alfa doses expected to be administered from baseline to week 48 of Study 250-201. Data in **C** and **D** are expressed as the mean ± 95% CI. Supplemental Tables 1 and 3 list the individual values for each data point and each patient.

Total HS and HS-NRE concentrations in CSF were measured weekly over the course of Study 250-201 Part 1 (Figure 3, A and B). For reference, HS and HS-NRE concentrations were measured in the CSF and plasma of nonaffected patients, and 95th percentile values (148 and 10 ng/mL, respectively) were used to identify elevated levels of these biomarkers. Patients 9001, 9002, and 9003 had respective total HS values of 255, 208, and 457 ng/mL and HS-NRE values of 48, 48, and 105 ng/mL at baseline in Study 250-201 Part 1. Tralesinidase alfa 30 mg administered i.c.v. reduced the levels of total HS and HS-NRE in both CSF and plasma in all 3 patients within weeks of dosing; however, only 300 mg tralesinidase alfa could sustain total HS normalization in all 3 patients for at least 6 weeks and HS-NRE normalization in 2 of the 3 patients. HS-NRE levels in the CSF of patient 9003 remained in the 20–28 ng/mL range, above the 95th percentile of nonaffected patients but close to the maximum value measured in unaffected CSF samples (18.6 ng/mL, Figure 3B) after 6 weeks of 300 mg tralesinidase alfa i.c.v. administration. There is no evidence that ADA interference could explain the HS-NRE levels observed in the CSF of patient 9003, considering the low level of neutralizing ADAs in the patient’s CSF.

During the course of Study 250-201 Part 2, HS-NRE levels in the CSF were normalized after the first 4 weeks of treatment to levels below the 10 ng/mL higher-end-of-normal cutoff (Figure 3C and Supplemental Table 1). Total HS levels in the CSF were similarly reduced after treatment with tralesinidase alfa (Supplemental Table 2). Of note, HS quantification was not affected by the presence of tralesinidase alfa in the sample because (a) the samples were collected prior to treatment and 1 week after the last treatment, and (b) tralesinidase alfa would not be functional extracellularly or at the CSF neutral pH. Patient 9003 was the only individual for whom the CSF levels of HS-NRE never normalized and consistently fluctuated between 15 and 25 ng/mL (Supplemental Table 1). Two other patients (patients 9017 and 9022) received only 17% and 57% of the intended dose of tralesinidase alfa and were therefore excluded from the data in Figure 3, C and D. Prior to treatment interruption, both of these patients had had CSF and total HS-NRE levels within the normal range (Supplemental Tables 1 and 2).

We observed significant correlations between total HS and HS-NRE levels in both CSF and plasma at different time points (Supplemental Tables 1–4). The reductions in HS and HS-NRE in both plasma and CSF were also significant as a result of tralesinidase alfa treatment (*P* < 0.0001). HS-NRE levels in plasma were significantly reduced to less than 40 ng/mL in participants after 4 weeks of administration of 300 mg tralesinidase alfa (Figure 3D). Six participants had plasma HS-NRE levels that fluctuated between 15 and 24 ng/mL during the 48-week study (Supplemental Table 3). In general, the concentrations were close to the maximum value assessed in plasma for nonaffected patients, i.e., 21.3 ng/mL (Supplemental Table 3). Total HS concentrations were also significantly reduced in plasma as a result of tralesinidase alfa administration (Supplemental Table 4).

### Weekly administration of tralesinidase alfa 300 mg resolves organomegaly.

**Figure 4:**
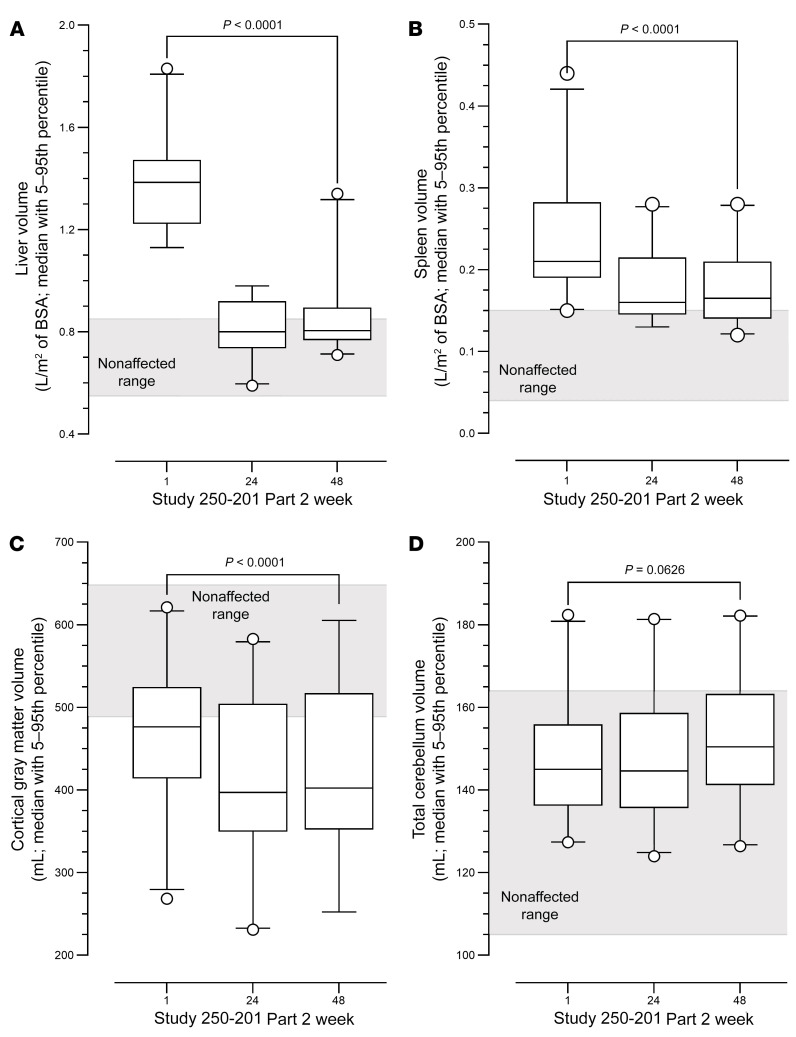
Changes in liver, spleen, cortical gray matter, and cerebellum gray matter volumes over the course of Study 250-201 Part 2. Liver (**A**), spleen (**B**), and brain subregions (**C** and **D**) were measured by MRI at week 1 (baseline) and weeks 24 and 48. Ranges for nonaffected patients were defined on the basis of previous publications (13, 14, 36, 37). MRI data were collected as described in Methods. Liver and spleen volumes in **A** and **B** were adjusted for BSA. Individual values in **C** and **D** for each participant at week 1 of Part 1 and weeks 1, 24, and 48 of Part 2 are listed in Supplemental Table 5. Boxes represent the 5th–95th percentiles with the median; dots represent values outside the 5th–95th percentiles. *P* values comparing week 1 (baseline) with week 48 were calculated using a 2-tailed, paired *t* test as determined by GraphPad Prism 9.3.1. *n* = 22 for liver and spleen; *n* = 19 and *n* = 20 for cortical gray matter and cerebellum, respectively.

Changes in liver and spleen volumes at weeks 1, 24, and 48 of Study 250-201 Part 2 are depicted in Figure 4, A and B; volumes are expressed as milliliters of tissue corrected for square meters of body surface area (BSA), which was calculated using the Mosteller method (12). Compared with the liver volumes for nonaffected children aged 2–10 years, estimated to be between 0.55 and 0.85 L/m^2^ (12, 13), the liver volumes for children with MPS IIIB ranged from 1.13–1.83 L/m^2^ BSA, for a mean value of 1.40 (median, 1.39) at the baseline visit for Study 250-201 Parts 1 and 2 (Figure 4A). Of the 4 patients with liver volumes of greater than 1.0 L/m^2^ of BSA at week 48, two appeared to have fast-progressing disease based on changes in both cortical gray matter volume (CGMV) and cognitive score, and 2 were the patients who received only 17% and 57% of the expected doses of tralesinidase alfa. By week 24 of Study 250-201 Part 2, the mean liver volume was reduced to 0.82 L/m^2^ (median, 0.80; range, 0.59–0.98), and by week 48, the mean value was 0.87 (median, 0.81; range, 0.71–1.34; Figure 4A). The reduction in liver volume was statistically significant (*P* < 0.0001, 2-tailed, paired *t* test) when comparing measurements at week 48 versus those at baseline.

At Study 250-201 Part 1 or Part 2 baseline, spleen volumes in children with MPS IIIB ranged from 0.15 to 0.44 L/m^2^ of BSA, for a mean value of 0.23 (median, 0.21), whereas normal spleen volumes for 2- to 10-year-old children were estimated to be between 0.04 and 0.15 L/m^2^ of BSA (13). By week 24 of Study 250-201 Part 2, the mean spleen volume decreased to 0.18 L/m^2^ (median, 0.16; range, 0.13–0.28), and this decrease was maintained through week 48 (median 0.17; range, 0.12–0.28; Figure 4B). The reduction in spleen volume was statistically significant (*P* < 0.0001, 2-tailed, paired *t* test) when comparing the volumes at week 48 with those at baseline.

### Tralesinidase alfa stabilizes brain atrophy in patients with MPS IIIB.

CGMV is a measure of the region of the brain most affected by atrophy in patients with MPS IIIB within the age range included in Study 250-201 (6). Patients with MPS IIIB enrolled in Study 250-201 had an average CGMV of 471 mL (median, 476; range, 268–621; Figure 4C). Nonaffected children would be expected to have a CGMV of 489 mL or greater (14). At week 48, eight patients had a CGMV within normal range; 5 patients had a CGMV between 473 and 485 mL or close to normal; 7 patients had a CGMV below normal, ranging from 384–455 mL; and the 2 oldest patients in the study, 116 and 118 months old at baseline, had CGMVs of only 268 and 343 mL, respectively (Supplemental Table 5).

Participants enrolled in Study 250-201 lost, on average, 58 mL (median 68 mL) of CGMV from baseline to week 24, but lost only 2 mL from week 24 to week 48, for a total loss of 60 mL (median, 63 mL) from week 1 to week 48 (*P* < 0.0001, 2-tailed, paired *t* test in both cases). Every participant in Study 250-201, except 2, lost CGMV during the course of Study 250-201 (Supplemental Table 5). One of the 2 participants who presented with an increase in CGMV was the youngest one, who gained 36 mL CGMV from baseline to week 24. Meanwhile, the same patients gained on average of 12 mL (median, 9.5 mL) in cerebral ventricle volume from baseline to week 24, but only 1 mL (median, –2 mL) from weeks 24 to 48 (Supplemental Table 5). The gain of 13 mL of cerebral ventricular volume over 48 weeks was slightly higher than the gain of 8 mL previously observed in the natural history study (6).

Nineteen of 22 patients treated with tralesinidase alfa had a total cerebellar volume within normal range at baseline (Figure 4D and ref. 6). Three patients had cerebellar volumes above the normal range at baseline. The mean cerebellar volume increased to 149 and 153 mL at week 24 and week 48, respectively, from 148 mL at baseline (Figure 4D). The increase in total cerebellar volume from week 1 to week 48 approached statistical significance (Figure 4D; *P* = 0.0626, 2-tailed, paired *t* test).

### Correlations between tralesinidase alfa exposure and cognitive decline trajectory and plasma HS-NRE levels.

**Figure 5:**
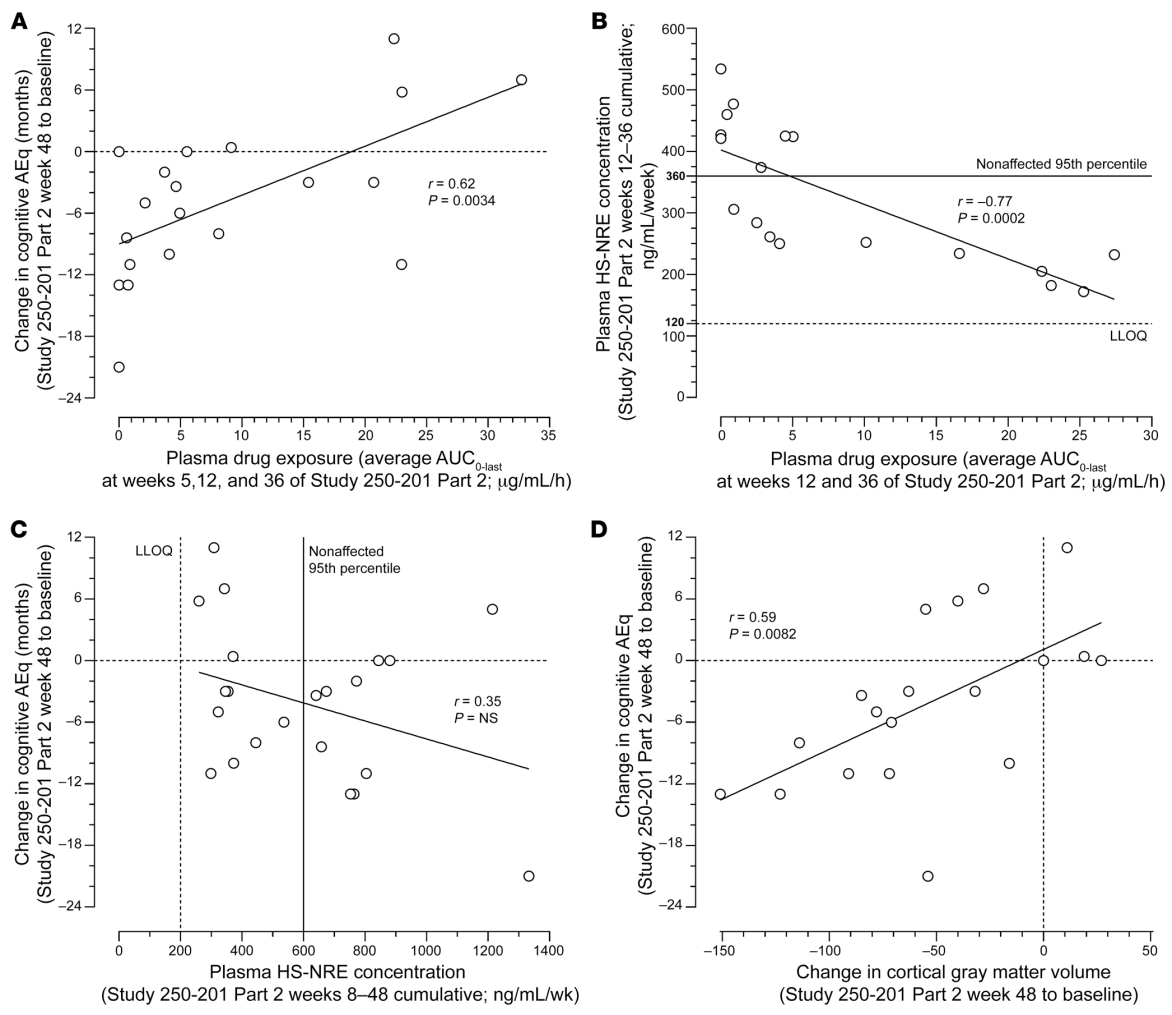
Correlation analyses of cognitive AEq scores, plasma drug exposure, plasma HS-NRE concentrations, and CGMVs over 48 weeks of tralesinidase alfa treatment. (**A**) Change in cognitive AEq score from Study 250-201 Part 2, week 1 to week 48, versus the average plasma drug exposure, i.e., AUC_0–last_, at weeks 5, 12, and 36 of Study 250-201 Part 2. (**B**) Plasma HS-NRE cumulative concentrations from weeks 8 to 48 of Study 250-201 Part 2 versus the average plasma drug exposure, i.e., AUC_0–last_, at weeks 5, 12, and 36 of Study 250-201 Part 2. the HS-NRE LLOQ was defined as 200 ng/mL/week, i.e., 5 ng/mL/week times 40 weeks, whereas the 95th percentile for nonaffected individuals was defined as 15 ng/mL/week times 40 weeks. (**C**) Change in cognitive AEq scores from Study 250-201 Part 2, weeks 1 to 48, versus plasma HS-NRE cumulative concentrations from weeks 8 to 48 of Study 250-201 Part 2. (**D**) Change in cognitive AEq scores from Study 250-201 Part 2, weeks 1 to 48, versus the change in CGMVs from Study 250-201 Part 2, weeks 1 to 48. Pearson’s *r* correlation and *P* values were calculated using GraphPad Prism 9.3.1. *n* = 20, *n* = 18, *n* = 22, and *n* = 19 for **A**–**D**, respectively.

A treatment duration of longer than 48 weeks will be needed to establish whether there is a clinically meaningful benefit for cognition related to treatment with tralesinidase alfa. However, to elucidate whether there is early evidence of an effect of tralesinidase alfa on disease trajectory in patients with MPS IIIB, we explored correlations between a change in cognitive AEq and plasma drug exposure, defined as AUC_0–last_, at weeks 5, 12, and 36 of Study 250-201 Part 2, HS-NRE biomarker levels, and CGMV over the 48-week study period. We noted a significant correlation between the change in cognitive AEq over the course of Study 250-201 Part 2 and average tralesinidase alfa exposure (AUC_0–last_) in plasma at weeks 5, 12, and 36 (Pearson’s *r* = 0.62, 2-tailed *P* = 0.0034, *n* = 20; Figure 5A). Three of the 4 patients with an average AUC_0–last_ of greater than 22 μg/mL/h had an increase of 5 months or more in AEq over 48 weeks, while 5 of the 6 participants with an AUC_0–last_ of less than 1 μg/mL/h lost between 8 and 21 months of AEq during the same period (Figure 5A).

A cumulative plasma HS-NRE concentration was calculated for each participant by integrating plasma HS-NRE concentrations measured every 4 weeks between weeks 12 and 36 of Study 250-201 Part 2. The average drug exposure, i.e., AUC_0–last_, in plasma at weeks 12 and 36 inversely correlated with cumulative plasma HS-NRE concentrations over the same period, i.e., the average of weeks 12 and 36 (Pearson’s *r* = –0.77, 2-tailed *P* = 0.0002, *n* = 18). Eight of the 12 patients with plasma drug exposure AUC_0–last_ of less than 6 μg/mL/h had plasma HS-NRE cumulative concentrations above the normal range, i.e., between 360 and 540 ng/mL/week (Figure 5B). Five of the 5 patients with HS-NRE cumulative concentrations between 170 and 240 ng/mL/week, i.e., within the normal range, from weeks 12–36, had plasma AUC_0–last_ of greater than 15 μg/mL/h. Patients with the highest plasma drug exposure were those with the lowest plasma HS-NRE concentrations, which were measured repeatedly between weeks 12 and 36 of Study 250-201 Part 2, and vice versa.

### Correlations of cognitive decline trajectory compared with plasma HS-NRE and cortical gray matter volume.

We also observed an inverse correlation between cumulative plasma HS-NRE concentrations and a change in cognitive AEq score from week 1 to week 48 of Study 250-201 Part 2 (Figure 5C). We noted a trend showing that 4 of 5 patients with a positive change in their AEq score from week 1 to week 48 had cumulative plasma HS-NRE concentrations within the normal range, i.e., between 260 and 380 ng/mL/week, from week 8 to week 48 (*r* = –0.35, *P* = NS, *n* = 22).

We observed a correlation between a change in the cognitive AEq score over the course of Study 250-201 Part 2 and a change in CGMV during the same period (Figure 5D; Pearson’s *r* = 0.59, *P* = 0.0082, *n* = 19). All 4 patients with change in CGMV of 0 mL or greater from week 1 to week 48 of Study 250-201 Part 2 had a change in AEq score of 0 or higher, while the patient who received only 17% of their intended dose of compound lost 54 mL in CGMV and had a loss of 21 months on the cognitive AEq scale.

## Discussion

In the present study, we show that, within weeks of weekly i.c.v. administration, 300 mg tralesinidase alfa could effectively compensate for NAGLU enzymatic activity as demonstrated by the normalization of HS and HS-NRE levels in both the CSF and plasma. Consequently, we observed resolution of hepatomegaly in most of the patients with MPS IIIB within 24 weeks of tralesinidase alfa administration. The presence of ADAs had no effect on HS normalization, hepatomegaly resolution, or the rate of cognitive decline. A longer treatment duration will be needed to fully evaluate the capacity of tralesinidase alfa to positively affect the lives of patients with MPS IIIB. The safety data for tralesinidase alfa (300 mg) administered weekly via i.c.v. infusion are in line with those for other ERTs, especially agents such as cerliponase alfa administered via i.c.v. dosing (15).

Our study of 22 patients with MPS IIIB, aged 25–118 months at baseline, established an effective dose of 300 mg tralesinidase alfa administered i.c.v. as necessary to achieve and sustain normalization of both total HS and disease-specific HS-NRE levels. The normalization of total HS and HS-NRE levels in the CSF provides evidence that tralesinidase alfa can be potentially beneficial to patients with MPS IIIB. While HS-NRE normalization in CSF is critical and a prerequisite for potential efficacy, the concentration of both tralesinidase alfa and the greater dynamic range of measurement of HS-NRE levels in plasma might be indicative of the efficacy of tralesinidase alfa in the CNS (Figure 5). We hypothesize that plasma drug exposure and HS-NRE levels may indirectly indicate how well the glymphatic system is working.

It is classically assumed that CSF flow is unidirectional and, therefore, that proteins directly administered in the CSF should be rapidly transported out of the brain and into the periphery (16). According to this model, i.c.v. delivery should result in little distribution of tralesinidase alfa in the brain. Our preclinical data have established that tralesinidase alfa can be effectively distributed throughout the brain of NAGLU-deficient mice and dogs, thereby preventing disease manifestations (10, 11). Data in the dog model of MPS IIIB demonstrated that HS-NRE levels in the CSF and brain correlate with each other; the normalization of HS-NRE levels in the CSF predicts benefits (11). Others have also recognized the value of CSF HS as a predictive marker of clinical efficacy (17); unfortunately, i.v. delivery of recombinant *N*-sulfoglucosamine sulfohydrolase resulted in no resolution of hepatosplenomegaly and no clear clinical benefits in a clinical study involving 6 patients with MPS IIIA. Although significant, the reduction in HS levels in the CSF of these patients was probably too slow and too little to translate into clinical benefits (17). Induction of ADAs has been suggested as an explanation for the lack of clinical efficacy (17); if true, it would make efforts to develop ERT for neurological disorders based on peripheral delivery, or the so-called molecular “Trojan horse” approach, challenging (16).

Our data favor an alternate explanation based on a recently identified pathway regarding the exchange of fluids and solutes from the CSF to the brain; the existence of the glymphatic system has now been established in human brain (18). The importance of a defective glymphatic system in the brain accumulation of toxic amyloid-β and tau proteins has been shown in animal models (19) and suggested as an explanation for the poor efficacy of immunotherapy in patients with Alzheimer’s disease (20). This dural lymphatic system and the active pumping of CSF into the periarterial spaces likely provide a way by which i.c.v.-dosed tralesinidase alfa can be effectively distributed from the CSF to the brain (21). Plasma hyperosmolality and the sleep cycle have been described as parameters that could modulate the delivery of macromolecules to the brain (22). Patients with the highest drug exposure and greatest sustained drug exposure, i.e., AUC_0–last_, in plasma were those who had the highest gain in AEq score from baseline to week 48 of Study 250-201 Part 2, and vice versa (Figure 5A); patients with the highest gain in AEq score also tended to have the lowest cumulative plasma HS-NRE concentrations from weeks 8 to 48 (Figure 5C). A significant inverse correlation was also observed between plasma drug exposure and cumulative plasma HS-NRE concentration (Figure 5B).

The use of both a sedative and a flushing solution could facilitate tralesinidase alfa distribution into the brain, possibly through the perivascular Virchow-Robin spaces (23, 24). The correlations between the change in cognitive AEq score and both plasma HS-NRE levels and plasma drug exposure (Figure 5, A and C) might reflect the ability of a given patient with MPS IIIB to effectively circulate macromolecules and metabolites from the brain to the periphery. A better preservation of their brain functionality and fluid exchanges with the periphery might provide some patients with MPS IIIB, in particular the youngest ones, with an advantage and greater efficacy of treatment with tralesinidase alfa. Over time, more patients might benefit from tralesinidase alfa treatment through a potentially slower but sustained improvement in their blood-CSF, brain-CSF, and brain-blood exchanges. We believe that tralesinidase alfa must be administered to the CSF via the i.c.v. route to circumvent the BBB so that sufficient drug concentrations can reach deep brain regions. However, plasma concentrations of drug and HS-NRE following i.c.v. treatment appeared to be indirect indicators of sufficient brain uptake and glymphatic function.

Advances in MRI procedures are enabling new and exciting opportunities to understand the exchange of fluids and solutes from the CSF to the periphery in patients (25, 26). For now, the cost and procedural difficulties make advanced MRI impractical for standardized medical practice. In the future, however, it would be interesting to understand the dynamics of CSF-plasma exchange in patients with MPS IIIB and its consequences on drug efficacy and resolution of pathology. Understanding of the dynamics of CSF-plasma exchange may inform and improve treatment procedures and increase the chance of success.

Our data show that ADAs had no impact on the PK and PD of tralesinidase alfa, unlike what has been reported by others (16, 17); most likely, direct i.c.v. administration of tralesinidase alfa explains this distinctive and advantageous observation. There was no correlation between plasma drug exposure at week 5 and serum ADA titers at week 4 (Pearson’s *r* = –0.29, *P* = NS). Neutralizing antibody testing for positive ADA samples in the CSF were low; less than 1:100 was the highest titer measured (data not shown), suggesting no effect of ADAs on drug exposure or efficacy in the brain tissue, the primary target organ. The majority of tralesinidase alfa–treated patients (19 of 22; 86%) had normalized HS and HS-NRE levels in both the CSF and plasma during the course of Study 250-201 Part 2, regardless of their ADA titers (Figure 1, C and D, and Figure 2, C and D), again arguing that ADAs have no impact on tralesinidase alfa function. Even if serum or CSF ADAs could interfere with plasma drug exposure and plasma HS-NRE clearance, there is no evidence that ADAs influenced the changes in CGMV during the course of Study 250-201 Part 2. ADAs clearly had no impact on the resolution of hepatomegaly (Figure 4A).

We believe that the initial accelerated loss of CGMV in the first 24 weeks of Study 250-201 Part 2, i.e., 58 mL on average, versus 22 mL for the same patients in natural history Study 250-901 (6), reflects an HS clearance out of the brain, whereas the average loss of only 2 mL from weeks 24–48 demonstrates the ability of tralesinidase alfa to stabilize brain atrophy, especially since 9 patients had higher CGMVs at week 48 than week 24 of Study 250-201 Part 2. One hypothesis is that HS clearance as a result of tralesinidase alfa treatment allows for the buildup of a denser dendritic network among neurons, which leads to stabilized, and perhaps increased, CGMV (27). Considering the importance of HS in neurogenesis, it would be nice to imagine that, by restoring a natural physiological cycle for HS, tralesinidase alfa could favor postnatal neuronal cell division or even promote the differentiation of neural stem cells, especially in the youngest patients (28).

The data presented here and in our natural history study (6) indicate that the preservation of CGMV is linked to the preservation of cognitive development. It is known that HS buildup in the lysosomes can cause neuronal cell death (29) and that brain atrophy, in turn, leads to cognitive loss. While normalizing biochemical function is likely a prerequisite for clinical efficacy, additional factors are expected to contribute to the best clinical outcomes, such as the patient’s age and degree of brain atrophy at treatment onset, the duration of treatment, and the fraction of the delivered dose taken up by the brain versus CSF drainage to the periphery. Although our data suggest favorable results, a longer treatment duration is needed to assess the long-term effects of tralesinidase alfa on cognition.

The present analysis has focused on the PK of tralesinidase alfa, the normalization of HS in the CSF and plasma following tralesinidase alfa administration, and its impact on hepatosplenomegaly and stabilization of brain atrophy. In addition, our data show a positive effect of tralesinidase alfa on cognitive function in patients with the highest plasma drug exposure and the lowest levels of HS in plasma. Yet, we recognize the limitations of the current study. In particular, the number of enrolled patients was limited because of the ultra-rare nature of the disease. The treatment duration was only 48 weeks for the great majority of the patients; however, our ongoing clinical study seems to confirm a long-term effect of tralesinidase alfa on cognitive and communication skills. Other long-term effects on sleep behavior and hearing and other quality-of-life measures are being monitored and will be reported in a future publication. Nonetheless, the analyses described in the current study can be applied to other forms of MPS and more broadly to other LSDs with neurological manifestations.

In conclusion, the present study demonstrates the ability of 300 mg tralesinidase alfa to normalize HS and HS-NRE levels in CSF, resolve hepatomegaly, and stabilize brain atrophy in patients with MPS IIIB. ADAs did not interfere with tralesinidase alfa efficacy. The safety profile of tralesinidase alfa is consistent with what is expected of a drug administered i.c.v. Plasma drug exposure and plasma HS-NRE levels may indirectly indicate glymphatic function, which may play a role in improved efficacy. Considering the severity of MPS IIIB, it would be unexpected for tralesinidase alfa to provide clinical benefits to every patient, at least in terms of cognitive function. However, the early effect observed in a few patients within only 48 weeks of treatment is very encouraging and warrants further evaluation. In light of the poor and eventually fatal prognosis and the absence of disease-modifying therapies, tralesinidase alfa has the potential to change the course of the disease in children with MPS IIIB.

## Methods

### Study designs.

Interventional study NCT02754076, i.e., Study 250-201, was a phase I/II open-label, dose-escalation (Part 1), and stable-dose (Part 2) study involving males and females between 1 and less than 11 years of age (i.e., 12 months and 132 months) with a confirmed diagnosis of MPS IIIB. Three patients, i.e., patients 9001, 9002, and 9003, were enrolled in Study 250-201 Part 1 and received weekly escalating doses, i.e., 30, 100, and 300 mg of tralesinidase alfa via an i.c.v. device such as the Ommaya reservoir and a catheter implanted into the lateral ventricle. Twenty-three patients, 19 of whom were rolled over from our natural history study (6), were enrolled in Study 250-201 Part 2 with the intent of receiving 48 weekly infusions of 300 mg tralesinidase alfa; 1 patient discontinued participation in the study after consent but prior to reservoir implantation, and 1 patient discontinued the study on the recommendation of the data monitoring committee (DMC) following a SAE of subdural hygroma associated with a subdural hematoma and increased intracranial pressure. In total, 23 patients were recruited in 6 different countries. Tralesinidase alfa was administered in a 10 mL solution of artificial CSF followed by flushing of the infusion line with a flushing solution. The composition of the artificial CSF was as follows: sodium phosphate monobasic monohydrate 0.4 mg/mL, sodium phosphate dibasic heptahydrate 0.19 mg/mL, sodium chloride 8.66 mg/mL, potassium chloride 0.22 mg/mL, magnesium chloride hexahydrate 0.16 mg/mL, and calcium chloride dihydrate 0.21 mg/mL.

The sample size was determined by the number of patients who were rolled over from Study 250-901 into Study 250-201. On the basis of the natural history data for untreated patients with MPS IIIA, 10 points of yearly decline in cognitive DQ score was assumed for the natural course of MPS IIIB, and the SD of the difference in yearly decline between pre- and post-treatment periods within a patient was assumed to be 10. Under these assumptions, a sample size of 20 patients was estimated to provide greater than 90% power to detect an 8-point difference in yearly decline between pre- and post-treatment periods, using a paired *t* test at a 5% significance level. A total of 21 patients completed Study 250-201; the patients were sedated, if needed, during the treatment procedure.

The primary objectives were to (a) evaluate the safety and tolerability of tralesinidase alfa administered to patients with MPS IIIB via an implanted i.c.v. reservoir and catheter, and (b) evaluate the impact of tralesinidase alfa on cognitive function as assessed by the DQ. Additional objectives were to (a) evaluate the impact of tralesinidase alfa on cognitive function as assessed by AEq score; (b) characterize the PK of tralesinidase alfa in CSF and plasma; (c) characterize the immunogenicity of tralesinidase alfa in CSF and serum; (d) evaluate the impact of tralesinidase alfa treatment on CSF, serum, and urine GAGs; and (e) evaluate the impact of tralesinidase alfa treatment on brain structure, liver size, and spleen size as assessed by MRI.

### Inclusion and exclusion criteria.

All study participants had deficient NAGLU enzyme activity and *NAGLU* gene mutations confirmed by genetic testing, and all presented with signs and symptoms consistent with MPS IIIB. Individuals were excluded from the study if they (a) had another neurological illness that may have caused cognitive decline; (b) received stem cell transplantation, gene therapy, or ERT for MPS IIIB; (c) received any investigational medication within 30 days prior to the baseline visit or were scheduled to receive any investigational drug during the course of the study; (d) presented with a medical condition or extenuating circumstance that, in the opinion of the investigator, might compromise the patient’s ability to comply with protocol requirements, the patient’s wellbeing or safety, or the interpretability of the patient’s clinical data; (e) participated in another natural history study; (f) had contraindications for neurosurgery; (g) had contraindications for MRI scans; (h) had a history of a poorly controlled seizure disorder; (i) were prone to complications from intraventricular drug administration, including patients with hydrocephalus or ventricular shunts; or (j) required ventilation support, except for noninvasive support at night.

### Cognition.

Cognitive function was measured using the nonverbal scales of the Bayley Scales of Infant and Toddler Development, 3rd Edition (BSID-III) (30) or the Kaufman Assessment Battery for Children, 2nd edition (KABC-II) (31); the choice of test was determined at study screening and for further visits using a predefined algorithm (Supplemental Appendix 1). AEq scores from published normative data were obtained from raw scores, and the DQ was derived by dividing the AEq by chronological age and then multiplying by 100. Use of AEq scores circumvents the “floor effect” of standardized scores when applied to children with severe cognitive impairment (32).

The BSID-III is a validated and standardized developmental tool comprising 5 domains (cognitive, language, motor, social-emotional, and adaptive functioning) intended to assess developmental function in children ages 1 to 42 months (30). Cognitive assessment is done individually by a qualified rater to capture the development of critical skills such as processing speed, problem solving, and play. Importantly, the cognitive assessments do not require the child to respond verbally, rendering this test particularly useful for assessing cognitive function in conditions such as MPS IIIB, in which expressive language might be limited. Raw scores, i.e., the numbers of correct responses, allow the generation of an AEq score and a DQ; these latter scores for the cognitive domain are presented hereafter. Mean raw scores were also analyzed, but the data are not presented here.

The KABC-II is a validated and standardized clinical psychological diagnostic test for assessing cognitive development (31). The subtests that comprise the KABC-II nonverbal index include conceptual thinking, face recognition, story completion, triangles, pattern reasoning, and hand movements. In addition to the nonverbal index subtests, the knowledge cluster subtests (riddles, expressive vocabulary, and verbal knowledge) were administered to patients who had language. As with the BSID-III, the raw scores associated with different ages allow for the generation of AEq scores and a DQ. Mean age equivalent scores were averaged over the administered domains; these scores for the nonverbal index are presented hereafter.

BSID-III or the KABC-II assessments were performed every 12 weeks in Study 250-201. Baseline, week 24, and week 48 visits involved 2 days of testing, with the cognitive, adaptive, and behavioral testing occurring on the first day (morning preferred), and MRI, lumbar puncture, and laboratory testing occurring on the second day to avoid confounding effects of sedation or general anesthetic administration.

### MRI assessments.

Head and abdominal MRIs were used to assess regional brain, liver, and spleen volumes at baseline, week 24, and week 48 of Study 250-201. Brain volumetric analysis was performed on 3D T1-weighted images using the FreeSurfer Image Analysis Suite, version 5.3 (Martinos Center, Harvard University) (33) as described previously (34, 35). This analysis yielded surface-based cortical parcellation and volume-based morphometric segmentation.

Brain volumes are noted without normalization, similarly to previous publications (6, 34). Regional brain volumes of nonaffected children (controls) measured by the same procedures and the same central reader were used for comparison (14).

Liver and spleen volumes were calculated by ICON Laboratory Services. Because liver and spleen volumes increased linearly compared with both age and BSA, these volumes were normalized to BSA, calculated using the Mosteller formula to allow for comparison among patients ranging from 2–9 years of age (12). Normal ranges for BSA-corrected liver and spleen volumes were estimated on the basis of the literature (13, 36, 37).

### Laboratory testing.

Tralesinidase alfa quantification in CSF and K2EDTA plasma was based on an electrochemiluminescence immunoassay (ECLA) technique using standard-bind, 96-well Meso Scale Discovery (MSD) streptavidin-coated plates. Samples were collected before dosing and 0.5, 4, 10, 24, 48, 72, 96, and 168 hours after drug administration at weeks 1 (baseline), 5, 12, and 36 of Study 250-201 Part 2. CSF samples were collected from the lateral ventricle through the i.c.v. port. Methods were validated, and sample analysis was conducted by Charles River laboratories.

ADAs were measured in the CSF and serum using an ECLA method with MSD technology. Samples were diluted in a Master Mix with an equal concentration of ruthenylated (sulfo-tagged) tralesinidase alfa and biotinylated tralesinidase in the wells of polypropylene plates. Detection of ADAs is based on the bivalent characteristics of the antibodies; ADAs bind to both sulfo-tagged and biotinylated molecules to form an antibody complex bridge. Samples are dispensed onto streptavidin-coated MSD assay plates to allow binding of biotinylated tralesinidase alfa to the streptavidin in the wells. Only the samples that contain ADAs bound to both the biotinylated and sulfo-tagged tralesinidase alfa generate an ECL signal. In the presence of tripropylamine, ruthenium produces a chemiluminescent signal that is triggered when voltage is applied. The signal produced is proportional to the amount of ADAs present. Endpoint titer determination estimates the relative levels of ADAs for those positive samples. ADA assays were validated and samples were run by ICON Laboratory Services.

Neutralizing antibodies were quantified in CSF using a cell-based assay that measured the ability of ADAs to interfere on CI-MPR–mediated uptake of tralesinidase alfa by the human Jurkat cell line. Tralesinidase alfa conjugated to Alexa 647 was monitored and quantified by flow cytometry using median fluorescence intensity (MFI) as the readout. The method was validated and samples were analyzed by Eurofins Pharma Bioanalytics Services US.

Levels of tralesinidase alfa–specific IgE were quantified using tralesinidase alfa covalently coupled to ImmunoCAP. After washing away nonspecific IgE, a β-gal–labeled mouse monoclonal antibody against human IgE was added to form a complex. After incubation, unbound enzyme anti-IgE was washed away, and the bound complex was incubated with the ImmunoCAP Development Solution containing 4-methylumbelliferyl-β-d-galactoside, a β-gal fluorogenic substrate. The fluorescence measured in the eluate is directly proportional to the concentration of drug-specific IgE in the patient sample. The method was validated and samples were analyzed by Viracor Eurofins Clinical Diagnostics.

Total HS and HS-NRE measurements in CSF and plasma were performed by ARUP Laboratories using a previously described method (38). The lower limit of quantification (LLOQ) of the assay was 5 ng/mL for HS-NRE and 100 ng/mL for total HS. Cutoff values for nonaffected patients’ CSF (*n* = 60) and plasma (*n* = 91) samples were established at 148 and 323 ng/mL for total HS and 10 and 15 ng/mL for HS-NRE in CSF and plasma, respectively. *NAGLU* genotyping and enzyme activity testing were performed by the Greenwood Genetic Center.

### Statistical analysis.

No corrections or carry-forwards were made for missing values. A 2-tailed, paired *t* test and linear correlation analyses were performed using GraphPad Prism 9.3.1 (GraphPad Software). *P* values of less than 0.05 were considered significant.

### Study approval.

Written informed consent from a parent or legal guardian and assent from the participant, if required, was obtained prior to conducting any study-specific assessments. This study was approved by the IRB affiliated with each individual clinical site: Children’s Hospital Oakland, Oakland, California, USA; Mackay Memorial Hospital-Taipei Branch, Taipei City, Taiwan; Great Ormond Street Hospital for Children NHS Foundation Trust – Great Ormond Street Hospital, London, United Kingdom; Universitätsklinikum Hamburg-Eppendorf (UKE), Hamburg, Germany; Fundación Cardio Infantil – Instituto de Cardiología, Bogotá, Colombia; Complexo Hospitalario Universitario de Santiago (CHUS) – Hospital Clínico Universitario, Santiago de Compostela, Spain; and Gazi Üniversitesi Tıp Fakültesi, Cocuk Sagligi ve Hastaliklari AD, Cocuk Beslenme ve Metabolizma ve Çocuk Genetik Hastaliklari BD, Gazi, Ankara, Turkey.

## Author contributions

AJS and SMM designed the clinical protocol. NM, AK, KVC, IO, FE, PH, MJDCL, MLC, SPL, SB, MC, and MS recruited participants and performed and supervised clinical practices. IN designed and performed the brain MRI analysis. B Kuca supervised the clinical study execution and was responsible for regulatory submissions. EZ, JK, and B Kaufman performed the data analysis. EZ and JK designed the data analysis and wrote the original draft of the manuscript. All authors reviewed and edited the manuscript.

## Supplementary Material

Supplemental data

Trial reporting checklists

ICMJE disclosure forms

## Figures and Tables

**Table 2:**
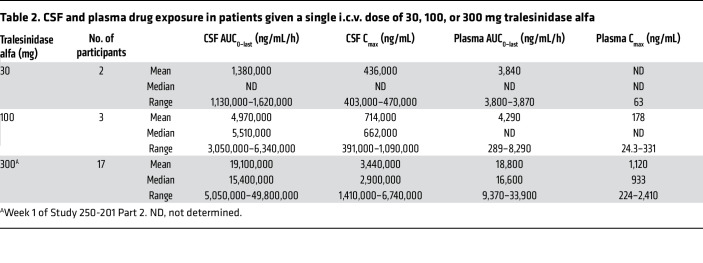
CSF and plasma drug exposure in patients given a single i.c.v. dose of 30, 100, or 300 mg tralesinidase alfa

**Table 1:**
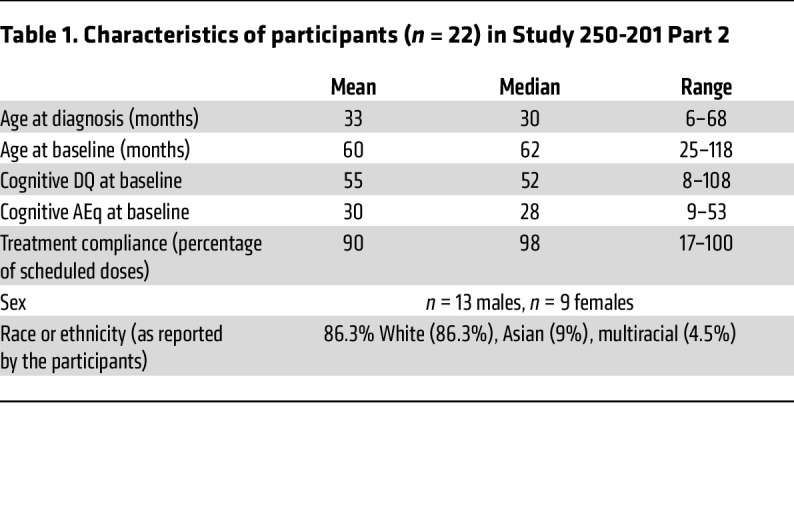
Characteristics of participants (*n* = 22) in Study 250-201 Part 2

## Version 1 11/22/2022

In-Press Preview

## Version 2 01/17/2023

Electronic publication
