# Supplementary material for: A phase I/II study on intracerebroventricular tralesinidase alfa in patients with Sanfilippo syndrome type B
Source: J Clin Invest. 2023 Jan 17;133(2):e165076. doi: 10.1172/JCI165076 (PMC9843052; doi:10.1172/JCI165076)
Supplement: Supplemental data [file jci-133-165076-s088.pdf]

Supplemental Table 1. CSF HS-NRE in individual subjects over the course of study 250-201[A]

Treatment Week	9001	9002	9003	9004	9005	9006	9007	9008	9009	9010	9011	9012	9013	9014	9015	9017	9018	9019	9020	9021	9022	9023
1 (Part 1)	48.0	48.1	104.6	Not applicable																		
1 (Part 2)	5.5	5.0	24.3	164.2	114.3	182.1	52.7	321.3	94.7	114.7	105.1	132.6	83.0	89.5	60.9	125.4	36.7	70.1	61.6	69.4	90.6	103.7
2		5.0			16.2		19.9			40.2	45.3	28.0	29.6		21.0	52.3			13.2	20.6	24.4	20.7
3	5.7	5.0	24.5	19.1		16.3	16.5	35.0	15.0	36.0	22.3	11.8	8.6	28.1	10.5	19.4	6.4	9.7	7.0	11.7		9.8
4	6.5	5.0	23.1	12.8	10.6	9.5	6.7		9.3	27.1	13.6	5.8	8.7	8.3	8.9	20.3		5.0	8.1	7.6	15.5	7.6
5	6.2	5.0	11.7	11.2	8.9		7.7	13.3	8.4	11.8	10.1	5.0	5.6	12.2	13.7	8.8		9.1	5.0	5.0	10.6	6.3
6	5.0	5.0				9.8	5.4	9.9	8.6	8.7	5.6	5.0	5.9	5.0	9.2	10.4		6.1		5.0	15.2	5.4
7	5.0	5.0	13.3	7.7	5.4	6.3	5.0	9.2	7.1	6.2	6.5	5.0	5.0	13.5	10.0	11.8		5.1	11.9	6.4	18.6	6.1
8	7.3	5.0	32.1	7.4	5.2	5.7	5.7	9.8	8.3	6.1	6.6	5.0	5.9	8.2	9.3	15.5		9.8	16.8	5.0	20.4	5.4
12	6.6	5.0	14.0	8.8	5.0	5.0	6.4	10.0	7.2	6.7	5.0	5.0	5.0	6.9	10.2			29.7	5.7	5.9	20.8	7.2
16	6.5	5.0	26.8	8.8	5.0	5.0	9.5	8.2	5.1	10.4	6.4	7.7	5.0	7.2	10.4		24.4	9.1	5.0	6.9		6.9
20	8.3	5.0	15.9	9.4	5.0	5.0	8.4	24.3	5.4	8.0	6.3		7.3	6.2	8.8		5.0	14.7		5.9		7.3
24	6.1	5.0	8.1	7.9	5.0		7.7	7.4	5.0	5.0	6.4	18.2	6.7	7.9	16.4		5.0	8.0	5.3	11.9		5.9
28		5.0	15.3		5.0		8.9	9.3		5.5	5.0	5.0		9.8	15.9		5.0	7.4		9.7	72.0	7.8
32	5.4	5.0	14.6	7.9	5.0	5.5	7.8	6.3	6.3	5.0	5.0	6.0		17.6			5.0	8.0	6.5	5.5	89.8	6.0
36	6.4	5.0	13.2	8.6	5.0	5.6	7.2	6.6	5.0	6.0	5.0	5.0	7.8	6.1	7.4		5.1	9.8	5.2	6.8		6.4
40		5.0	15.4	6.5	5.0	5.0	7.8	5.7	5.2	5.4		7.7	5.0	7.7	5.9		5.0	7.5	6.4	8.6		6.8
44	7.6	5.0	15.2	5.0	5.0	5.7	6.6	6.4	5.4	6.5	7.4	27.4	5.9	5.2	7.0		5.7	7.8	5.3	6.3	34.6	8.3
48	5.2	5.0	9.1	5.0	5.0	6.6	5.4	5.8	5.0	11.4	6.3	6.1	6.1	6.2	8.3		6.0	8.1	6.9	5.9	37.1	11.5

[A]Values in ng/mL; LLOQ = 5.0 ng/mL; non-affected subjects 95%ile = 10.0 ng/mL

Supplemental Table 2. CSF total HS in individual subjects over the course of study 250-201[A]

Treatment Week	9001	9002	9003	9004	9005	9006	9007	9008	9009	9010	9011	9012	9013	9014	9015	9017	9018	9019	9020	9021	9022	9023
1 (Part 1)	254.6	207.6	457.3	Not applicable																		
1 (Part 2)	100.0	100.0	148.3	567.7	508.8	529.9	217.0	752.0	380.8	393.4	385.5	493.3	368.2	337.9	235.2	368.3	216.6	261.0	273.3	410.8	439.4	335.3
2		100.0			128.2		127.7			188.2	188.3	157.6	164.1	135.6	125.9	174.9	100.0		114.7	142.1	147.0	114.1
3	100.0	100.0	137.7	124.6		103.9	104.4	146.7	110.5	195.9	127.3	104.0	112.1	100.0	101.8	105.5		100.0	100.0	103.3		100.0
4	100.0	100.0	144.8	100.0	100.0	100.0	100.0		100.0	167.7	108.0	100.0	100.0	105.2	100.0	116.0		100.0	100.0	100.0	111.7	100.0
5	100.0	100.0	107.4	100.0	100.0		100.0	107.8	100.0	100.0	100.0	100.0	100.0	100.8	104.5	100.0		191.7		100.0	100.0	100.0
6	100.0	100.0				100.0	100.0	100.0	100.0	100.0	100.0	100.0	100.0	121.7	103.6	100.0		100.0	100.0	100.0	109.9	100.0
7	100.0	100.0	106.2	100.0	100.0	100.0	100.0	100.0	100.0	100.0	100.0	100.0	100.0	101.5	104.0	108.5		100.0	131.6	100.0	137.0	100.0
8	100.0	100.0	217.0	100.0	100.0	100.0	100.0	105.0	100.0	100.0	100.0	100.0	100.0	110.7	100.0	128.2		103.3	145.3	100.0	167.1	100.0
12	100.0	100.0	108.2	106.6	100.0	100.0	100.0	115.9	100.0	100.0	100.0	100.0	100.0		112.6			165.1	100.0	100.0	160.8	100.0
16	100.0	100.0	150.2	114.1	100.0	100.0	100.0	118.9	100.0	113.2	100.0	140.2	100.0	100.0	100.0		223.2	112.9	100.0	100.0		100.0
20	100.0	100.0	126.6	100.0	100.0	100.0	100.0	132.4	100.0	100.0	100.0		100.0	100.0	100.0		100.0	116.7		100.0		100.0
24	100.0	100.0	100.0	100.0	100.0		100.0	100.0	100.0	100.0	100.0	151.0	100.0	100.0	146.0		100.0	100.0	100.0	117.5		100.0
28		100.0	116.1		100.0		100.0	101.4	100.0	100.0	100.0	100.0		133.5	112.8		100.0	100.0		106.0	374.3	100.0
32	100.0	100.0	119.8	100.0	100.0	100.0	100.0	100.0	108.3	100.0	100.0	100.0		195.0			100.0	100.0	100.0	100.0	352.0	100.0
36	100.0	100.0	118.1	100.0	100.0	100.0	100.0	100.0	100.0	100.0	100.0	100.0	100.0	100.0	100.0		100.0	100.0	100.0	100.0		100.0
40		100.0	128.3	100.0	100.0	100.0	100.0	100.0	100.0	100.0		111.6	100.0	100.0	100.0		100.0	100.0	100.0	125.2		100.0
44	100.0	100.0	120.4	100.0	100.0	100.0	100.0	142.5	100.0	100.0	126.9	158.2	100.0	100.0	100.0		100.0	100.0	100.0	101.6	170.5	100.0
48	100.0	100.0	100.0	100.0	100.0	100.0	100.0	100.0	100.0	118.2	104.5	100.0	100.0	100.0	100.0		100.0	100.0	100.0	100.0	183.7	100.0

[A]Values in ng/mL; LLOQ = 100.0 ng/mL; non-affected subjects 95%ile = 148.0 ng/mL

Supplemental Table 3. Plasma HS-NRE level in individual subjects over the course of study 250-201[A]

Treatment Week	9001	9002	9003	9004	9005	9006	9007	9008	9009	9010	9011	9012	9013	9014	9015	9017	9018	9019	9020	9021	9022	9023
1 (Part 1)			71.7	Not applicable																		
1 (Part 2)			18.6	69.8	66.6	80.1	85.0	117.5	98.0	55.7	71.0	48.0	90.4	57.2	67.4	78.8	45.5	62.1	27.3	61.1	71.5	
2																						
3																						
4	15.6	8.8	25.7		11.7	8.8	11.3		10.4	10.9	8.7	13.8	47.2	11.8	20.8	14.0	8.3	9.0	6.7	8.8	8.5	
5	24.6	11.5	19.2	9.1	11.2	7.7	8.5	11.9	10.3	13.5	7.3	11.4	8.2	12.8	20.3	12.4				7.2	11.1	
6																					6.8	
7																						
8	21.0		8.3	7.7			13.1	11.9	12.1	10.5	7.8	8.6	8.8	13.8	24.0	14.4		9.8	10.9		12.6	17.7
12	23.9	9.3	21.6	13.9	6.6	9.7	18.3	16.1	12.0	15.1	6.6	9.5	9.7	14.9	20.4			13.8	7.0	15.0	13.3	11.4
16	23.1		22.2	16.0	12.0	8.0	17.8	17.0	18.6	11.4	5.6	10.3	12.5	14.4	19.2		10.5	15.4	11.9			
20	22.5	8.0	22.1	16.6	7.1	6.0	16.9	22.5		11.6	7.0	7.3	5.6		14.1			23.5	10.1			
24	23.7	7.2	20.9	19.6	5.8	9.6	21.5	20.7		10.5	8.2	15.3	14.0		16.3	20.9		20.4	8.2			
28	24.5	9.7	22.8		8.4		15.5	20.2		10.5	8.2	13.8			13.3	50.2	14.4	15.8	8.7		31.2	13.6
32		14.0	22.0	17.2	6.0	9.3	23.2	20.5	18.9	8.7	6.9	8.7		21.5	21.5	51.7	12.0		11.0	9.5	42.4	13.9
36		11.8	25.3	24.2	5.9	8.0	22.0	20.5	12.5	10.2	7.6	5.9	5.0	16.1	23.3	45.7	11.8	18.4	7.7	9.9		11.8
40		8.8	27.2	22.1	6.8	7.1	17.6	25.1	24.6		8.6	6.3	6.0	16.3	19.1	41.5	10.1	16.1	7.5	9.6		12.4
44	15.3	8.7	22.0	27.2	7.0	9.5	18.3	23.2	22.6	10.9	9.1			13.9	23.0		9.7	16.3			41.7	15.8
48	15.4	8.5	20.0	23.9	5.6	11.1	21.4	18.6		13.0	6.3	8.2	9.5	13.5	21.6	42.3	10.3	13.4	7.9		35.3	17.3

[A]Values in ng/mL; LLOQ = 5.0 ng/mL; non-affected subjects 95%ile = 15.0 ng/mL

Supplemental Table 4. Plasma total HS in individual subjects over the course of study 250-201[A]

Treatment Week	9001	9002	9003	9004	9005	9006	9007	9008	9009	9010	9011	9012	9013	9014	9015	9017	9018	9019	9020	9021	9022	9023
1 (Part 1)			615.1	Not applicable																		
1 (Part 2)			264.7	629.1	652.9	765.8	815.3	984.8	928.5	658.9	780.6	575.7	548.7	756.1	692.2	712.3	457.8	603.9	302.3	536.7	810.6	
2																						
3																						
4	230.6	233.3	356.1		205.2	225.8	212.6		244.4	291.9	218.4	274.2	227.7	273.2	316.4	305.5	196.5	193.2	129.9	169.8	291.0	
5	287.2	263.4	302.9	174.2	240.8	182.4	189.0	244.6	221.5	308.5	186.0	233.0	227.7	265.9	286.5	246.0	180.7			152.5	287.3	
6																					100.0	
7																						
8	283.1		182.6	149.3			231.1	223.4	296.2	246.5	190.7	195.4	180.2	270.7	286.7	289.5		194.3			295.4	286.5
12	306.4	237.5	372.5	207.9	145.8	197.4	271.8	267.3	228.4	306.9	173.4	171.2	190.0	255.7	260.7			228.3	144.0	231.4	246.1	205.8
16	263.1		342.6	218.6	267.0	179.4	292.9	292.4	314.0	237.8	154.6	202.3	140.9	257.8	259.3			266.1	155.3			
20	322.3	197.3	355.0	230.7	155.0	127.5	266.3	347.4		228.2	165.5	179.7	165.0		248.1			337.8	145.4			
24	284.3	214.7	304.4	239.5	130.1	199.4	282.8	266.9		241.6	157.4	222.9	263.4		231.2	443.9		310.5	140.9			
28	286.9	215.0	371.1		169.9		262.3	292.1		220.6	145.0	253.5			288.8	507.5	215.8	244.9	165.5		465.5	258.7
32		342.5	310.0	241.2	123.9	180.6	283.2	279.1	331.2	213.5	145.3	178.9		314.2	319.9	526.8	230.2		203.0	188.3	599.5	284.5
36		283.4	377.0	277.5	147.9	184.2	257.5	304.0	284.8	226.4	143.2	152.2	116.2	288.4	307.5	481.0	220.5	262.1	143.6	201.7		242.0
40		217.5	445.1	263.7	140.5	184.5	259.9	338.9	375.7		136.1	165.4	136.3	260.7	318.0	437.9	220.2	226.9	148.2	203.4		252.5
44	243.5	258.4	370.8	276.2	126.7	184.8	273.1	309.0	321.2	209.4	128.3			259.1	370.9		177.5	258.1			661.9	274.5
48	229.9	221.0	283.3	305.4	136.6	216.2	279.1	285.9		197.0	126.4	166.6	150.2	247.8	405.5	566.3	201.7	283.1	137.0		455.5	287.7

[A]Values in ng/mL; LLOQ = 100.0 ng/mL; non-affected subjects 95%ile = 323.0 ng/mL

Supplemental Table 5. Cortical grey matter, cerebellum and cerebral ventricle volumes in individual subjects over the course of study 250-201

Subject	Age at baseline (months)	Cortical grey matter (mL)				Cerebellum (mL)				Cerebral ventricles (mL)			
		Week 1 Part 1	Week 1 Part 2	Week 24	Week 48	Week 1 Part 1	Week 1 Part 2	Week 24	Week 48	Week 1 Part 1	Week 1 Part 2	Week 24	Week 48
9001	127	268	253	247	252	161	158	157	155	105	137	134	134
9002	40	532	487	490	460	149	149	160	159	51	63	75	84
9003	46	621	565	551	592	154	159	157	162	26	21	22	20
9004	63		384	231	261		164	170	172		83	48	42
9005	59		585	547	545		182	181	182		24	39	35
9006	25		506	542	517		131	137	141		36	39	31
9007	46		486	397	334		147	149	144		55	85	65
9008	64		492	414	401		141	139	138		27	34	36
9009	93		478	384	393		143	134	139		38	58	54
9010	64		399	331	285		127	124	126		50	83	121
9011	47		439	352	367		151	142	142		25	35	32
9012	69		586	518	605		172	180	180		15	24	20
9013	64		415	312	352		172	164	166		28	87	128
9014	62		422	358			142	145	150		23	22	29
9015	71		412	405			154	152	160		39	49	40
9017	43		475	382	421		152	157	164		35	30	28
9018	69		590	583	574		138	145	141		22	39	40
9019	53		455	433	423		131	133	132		55	70	70
9020	118		343	347			128	135			23	29	
9021	44		480	391	402		129	134	78		48	64	63
9022	61		522		467		142		151		14		20
9023	31		473	409	402		143	142	149		64	99	92

Appendix 1.

Algorithm for the Choice of Cognitive Test (BSID-III vs KABC-II)

Each subject is designated to one of the testing instruments (KABC-II or BSID-III) at Screening. It is hoped that the designated test will remain appropriate at each visit for the full trial duration. However, it is possible that a subject designated to KABC-II may demonstrate disease progression and lose the ability to perform the KABC-II, necessitating a switch to the BSID-III. Conversely, it is possible that a younger subject may outgrow the BSID-III and need to switch to the KABC-II in order to measure her/his higher functioning. The guidelines for switching tests are as follows:

- For subjects designated to the KABC-II test, testing at each visit begins with the KABC-II Triangles subtest.

If the subject successfully performs 2 or more items, the KABC-II will continue to be used during the current visit and at the subsequent visit.

If the subject cannot successfully perform 2 or more items on the Triangles subtest, the rater should try one other nonverbal subtest of the KABC-II; if the subject successfully performs 3 or more items on the subtest, the KABC-II should continue to be used for the current visit and for the subsequent visit. If not, the KABC-II will be discontinued, and the BSID-III will be administered to the subject at this visit and at the subsequent visit.

- "For subjects designated to the KABC-II test, if the subject achieves the lowest age equivalent score (< 36 months) on any nonverbal subdomain AND has all other nonverbal subdomain scores ≤ 42 months, the subject will be switched to the BSID-III at the subsequent visit.

- “For subjects designated to the KABC-II test, if the subject achieves the lowest age equivalent score (< 36 months) on any nonverbal subdomain AND has at least one other nonverbal subdomain score > 42 months, the subject will continue to be tested with the KABC-II at the subsequent visit.”
- For subjects designated to the BSID-III test, if the subject successfully achieves the highest age equivalent score on the Cognitive domain (> 42), the subject will be switched to the KABC-II at the subsequent visit.
